# Luteolin as a multifaceted immunomodulator: insights into its effects on diverse immune cell populations and therapeutic implications

**DOI:** 10.3389/fimmu.2025.1621367

**Published:** 2025-10-14

**Authors:** Xiaolan Wang, Junbo Zhao, Ying Li, Sujun Gao, Long Su

**Affiliations:** ^1^ Department of Hematology, The First Hospital of Jilin University, Changchun, China; ^2^ Key Laboratory of Hematology Precision Medicine of Jilin Province, The First Hospital of Jilin University, Changchun, China; ^3^ Department of Hematology, Changchun Central Hospital, Changchun, China

**Keywords:** luteolin, modulation, T cells, natural killer cells, dendritic cells, macrophages, granulocytes

## Abstract

Luteolin, a natural flavonoid, exerts broad immunomodulatory effects across multiple immune cell populations, positioning it as a promising candidate for treating inflammatory diseases, infections, and cancer. This review synthesizes current evidence on luteolin’s effects on T cells, natural killer (NK) cells, dendritic cells (DCs), macrophages, neutrophils, eosinophils, and basophils. Luteolin promotes the differentiation of regulatory T cells (Tregs) and suppresses pro-inflammatory T helper 17 (Th17) and Th2 responses, thereby restoring immune balance in sepsis, allergies, and autoimmunity. In macrophages, it skews polarization toward the anti-inflammatory M2 phenotype via the signal transducer and activator of transcription 3 (STAT3)/STAT6 and peroxisome proliferator-activated receptor γ (PPARγ) pathways, while inhibiting nuclear factor-κB (NF-κB) and NOD-like receptor family pyrin domain-containing 3 (NLRP3) inflammasome activation. Neutrophil functions are dampened by reduced oxidative stress, adhesion molecule expression, and induction of apoptosis. Luteolin may enhance NK-cell cytotoxicity and DC-mediated antigen presentation while curbing eosinophil and basophil activation in allergic disorders. Despite preclinical successes, future research should prioritize mechanistic insights, structural optimization, and clinical translation to unlock luteolin’s full therapeutic potential.

## 1 Introduction

**Figure 1:**
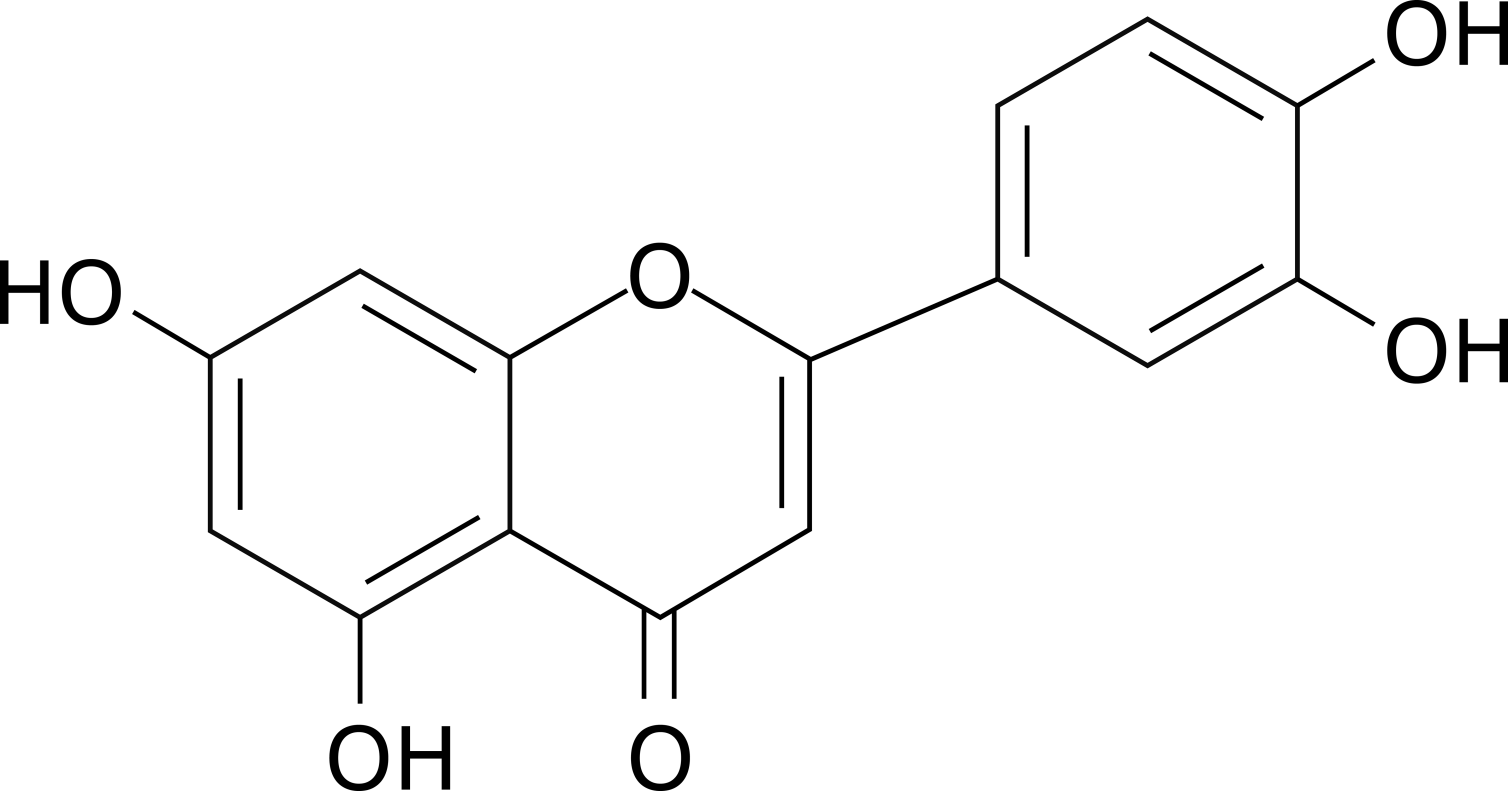
The chemical structural formula of luteolin.

The immune system is a dynamic network of cells and molecules that safeguards host health by orchestrating defenses against pathogens and maintaining tissue homeostasis. However, dysregulation of immune responses contributes to the pathogenesis of inflammatory disorders, autoimmune diseases, and cancer (1–3). Natural products, particularly flavonoids, have emerged as promising candidates for immunomodulation due to their ability to target multiple signaling pathways with minimal toxicity (4, 5). Among them, luteolin is a naturally occurring flavonoid compound with the molecular formula C_15_H_10_O_6_ (**Figure 1**). Structurally, it belongs to the flavone subclass, characterized by a 2-phenylchromen-4-one backbone substituted with hydroxyl groups at positions 3’, 4’, 5, and 7 (6). This configuration confers distinct physicochemical properties, including poor aqueous solubility and high solubility in organic solvents (7). First isolated in 1829 as pure luteolin from *Reseda luteola*, it was later identified in various plant species such as celery, thyme, rosemary, and chamomile (8). Its yellow pigmentation contributed to its historical use in textile dyeing, but the bioactive potential of luteolin was not fully explored until the 20^th^ century. Biologically, luteolin exhibits pleiotropic effects—such as antitumor, anti-infection, neuroprotection, and cardiovascular protection—primarily attributed to its antioxidant and anti-inflammatory properties (9, 10). Luteolin scavenges reactive oxygen species (ROS) and inhibits pro-inflammatory enzymes like cyclooxygenase-2 (COX-2) and inducible nitric oxide synthase (iNOS) (11). Additionally, it modulates immune responses by regulating neutrophil activation, macrophage polarization, and cytokine secretion (12–14). Recent studies highlight its roles in apoptosis induction and cell cycle arrest in cancer cells, suggesting potential anticancer activity (15). It also interacts with signaling pathways such as nuclear factor-κB (NF-κB), mitogen-activated protein kinase (MAPK), and phosphatidylinositol 3-kinase (PI3K)/protein kinase B (AKT), which are critical for inflammation and oncogenesis (15). Research advancements in the past decade have expanded luteolin’s broad-spectrum effects on immune cells (16–18). Previous reviews have documented the antitumor activity (15, 19, 20), neuroprotection (21–23), modulation of metabolic diseases (24–26), cardiovascular protection (27–29), and anti-infective activities (30–32) of luteolin. Moreover, while a previous review addressed the immunomodulatory effects of luteolin on specific immune cell types (e.g., T cells, macrophages, neutrophils, and dendritic cells), its focus was limited to inflammatory skin diseases (33). Similarly, prior reviews of other flavonoids have focused on a limited range of specific disorders (34–36). Thus, a comprehensive review of luteolin’s modulatory effects across a broader spectrum of heterogeneous immune cell populations (e.g., basophils and eosinophils) and in diverse disease settings remains underexplored in the current literature. This review synthesizes current understanding of the immunomodulatory effects and mechanisms of luteolin across diverse immune cell populations—including T cells, natural killer (NK) cells, dendritic cells (DCs), macrophages, neutrophils, eosinophils, and basophils—and discusses its therapeutic potential in inflammatory and immune-related disorders (**Table 1**, **Supplementary Table 1**).

**Table 1:** The impact of luteolin and its derivatives on multiple immune cells in different animal models.

Inducers	Models	Luteolin or derivatives	Impact on different immune cells	ECR *in vitro*
CLP (39)	Mouse ALI	20 mg/kg one hour prior puncture for once by ip	·Promote M2 macrophage polarization ·Enhance Treg differentiation ·Reduce neutrophil infiltration	NA
LPS (78)	Mouse ALI	18, 35, or 70 μmol/kg 30 minutes before LPS injection by ip	·Inhibit neutrophil activation and infiltration	NA
LPS (12)	Mouse ALI	70 μmol/kg five minutes after intratracheal LPS injection by ip	·Attenuate neutrophil chemotaxis and respiratory burst	0.3−10 μmol/L
OVA (89)	Mouse asthma	10, 50, or 100 mg/kg for 7 doses with a 12-hour interval by gavage^*^	·Inhibit eosinophil infiltration ·Inhibit Th2 cell differentiation	NA
OVA (90)	Guinea pig asthma	30 mg/kg one hour prior to OVA, and 6, 12 and 20 h later by gavage	·Decrease total leukocyte recruitment ·Decrease eosinophil recruitment	NA
OVA (40)	Mouse airway inflammation	20 mg/kg for six weeks by gavage	·Suppress eosinophil infiltration ·Increase Treg levels ·Reduce B cells, T cells and MDSCs	10 μg/mL
Bleomycin (83)	Mouse lung fibrosis	10 mg/kg during the indicated time period by gavage	·Reduce neutrophil infiltration	NA
OVA (41)	Mouse AR	2.5, 12.5, or 25 mg/kg for 14 days by gavage from the 22^nd^ day of experiment	·Ameliorate Th1/Th2 imbalance ·Alleviate Tregs/Th17 imbalance	NA
OVA (42)	Mouse AR	5 or 10 mg/kg 30 minutes prior OVA for 11 days by gavage	·Alleviate Tregs/Th17 imbalance	NA
OVA (43)	Mouse AR	0.1, 1, or 10 mg/kg 30 minutes prior OVA for 14 days by ip	·Ameliorate Th1/Th2 imbalance	NA
House dust mite (88)	Mouse AR	10 or 30 mg/kg for 27 days by ip	·Decrease Th1 and Th17 cells ·Reduce infiltration of eosinophils	2.5−10 μg/ml
DSS (16)	Mouse colitis	5 mg/kg for 7 days by gavage ^<ns/>^	·Decrease Th1, Th2, and Th17 cells ·Reduce neutrophil infiltration	20 μg/ml
DSS (14)	Mouse colitis	12, 25 or 50 mg/kg for 17 days by gavage	·Promote M2 macrophage polarization	6.25−25 μmol/L
B16F10 cells (47)	Tumor-bearing mice	10 mg per mouse injected intramuscularly on the first and seventh days with irradiated B16F10 cells	·Activate APCs and increase antigen presentation ·Activate Th1, Th2, and CD8^+^ T cells ·Decrease levels of Tregs	40 μg/mL
A549 and HCT116 cells (48)	Tumor-bearing mice	25 or 50 mg/kg for 28 days by ip	·Promote CD8^+^ T cell proliferation, activation and cytotoxicity	40 μmol/L
Lewis cells (51)	Tumor-bearing mice	20 mg/kg for 18 day by ip when the tumor volume reached 100 mm^3^	·Enhance T-cell-mediated killing	10−80 μmol/L
H22 cells (49)	Tumor-bearing mice	50, 100, or 200 mg/kg for 28 days by gavage	·Enhance T cell activation, chemotaxis, and function ·Sustain a high ratio of CD8^+^ T lymphocyte ·Restore cytotoxicity of CD8^+^ T lymphocytes	NA
BCG (50)	BCG-immunized mice	5 mg/kg from one day after BCG injection for 20 days by ip	·Increase CD4+ and CD8+ T cells ·increase central memory T cells	NA
MT (52)	Mouse MT infection	5 mg/kg starting from 15 days after MT infection during the indicated time period by ip	·Increase total splenocytes and T cells ·Up-regulate CD44 and CD69 expression on T cells ·Increase central memory T cells ·Expand and activate NK and NKT cells	NA
LPS (85)	Mouse periodontitis	10, 30 or 100 mg/kg one hour after LPS injection for 14 days by gavage	·Inhibit neutrophil infiltration	NA
Ligature (71)	Rat periodontitis	30 μL (50 μM) every 48 hours for four weeks by gingival injection	·Enhance M2 macrophage polarization	20 μmol/L
TSH receptor A subunit (17)	Mouse Graves disease	30 mg/kg for 28 days (administration route was not mentioned)	·Promote Treg differentiation ·Restore Tfh/Tfr and Tfh/Treg balances	NA
Imiquimod cream (13)	Mouse psoriasis	50 mg/kg for 8 days by ip	·Alleviate Th1/Th2 and Th17/Tregs imbalance	50 μmol/L
MRL/lpr mice (68)	Mouse lupus nephritis	10 or 40 mg/kg during the indicated time period by gavage	·Inhibit HIF-1α pathway in macrophages	5−20 μmol/L
LPS (70)	Mouse liver injury	30 or 60 mg/kg once by ip	·Inhibit macrophage recruitment	NA
CFA (77)	Mouse arthritis	50 mg/kg before intraplantar CFA injection by ip	·Ameliorate neutrophil infiltration	3−30 μmol/L
Rabbit IgG (81)	Mouse pemphigoid	1 mg per mouse during experiment period by ip	·Block respiratory burst in granulocytes	50−200 μg/mL

^*^Luteolin-7-O-glucoside was used in this study; ECR, effective concentration ranges; GLM was used in this study; CLP, cecal ligation puncture; LPS, lipopolysaccharides; OVA, ovalbumin; DSS, dextran sulfate sodium; ALI, acute lung injury; AR, allergic rhinitis; BCG, Bacillus Calmette-Guérin; MT, mycobacterium tuberculosis; CFA, Complete Freund’s adjuvant; ip, intraperitoneal injection; Tregs, regulatory T cells; MDSCs, myeloid-derived suppressor cells; APCs, antigen-presenting cells; NA, not applicable.

## 2 The effects of luteolin on T cells

T cells, a fundamental component of the adaptive immune system, are pivotal in orchestrating immune responses. They recognize specific antigens, differentiate into distinct subsets, and execute functions crucial for maintaining immune balance and protecting the body against pathogens and tumors (37). Luteolin exerts diverse effects on T cells, which can be categorized based on the specific cellular and molecular alterations it induces. These effects have been investigated in multiple studies, providing insights into their potential applications in various physiological and pathological conditions.

### 2.1 Modulation of T cell subsets

T cell subsets play distinct roles in the immune response, and their balance is crucial for immune homeostasis. Luteolin has been shown to affect the differentiation and proportions of T cell subsets (38–41). It was reported that luteolin promotes the differentiation and function of regulatory T cells (Tregs) (38, 41). In a cecal ligation and puncture (CLP)-induced acute lung injury (ALI) mouse model, luteolin alleviated lung injury, suppressed uncontrolled inflammation, and upregulated levels of interleukin-10 (IL-10) in serum and bronchoalveolar lavage fluid (BALF). It increased the frequency of CD4^^+^^CD25^^+^^Foxp3^^+^^ Tregs in peripheral blood and in splenic mononuclear cells of ALI mice. *In vitro*, luteolin significantly induced the differentiation of Tregs (38). Zhang et al. also found that, in a CLP-induced ALI mouse model, luteolin activated Tregs to promote IL-10 expression and alleviated caspase-11-dependent pyroptosis in sepsis-induced lung injury. Depleting Tregs reversed the beneficial effects of luteolin on lung injury, indicating the importance of Tregs in the luteolin-mediated protective effect (39). Kim and colleagues demonstrated that luteolin increased the number of CD4^+^CD25^+^ Tregs *in vitro*, with elevated levels of transforming growth factor-β1 (TGF-β1) and Foxp3 messenger ribonucleic acid (mRNA) expression, and the transfer of these Tregs into ovalbumin (OVA)-sensitized mice (a murine model of airway inflammation) reduced airway hyper-responsiveness, eosinophil recruitment, and T helper 2 (Th2) cytokine expressions, and increased interferon-γ (IFN-γ) production (40). Luteolin can also regulate the Th17/Treg balance. In allergic rhinitis (AR) mouse models, luteolin restored the Th17/Treg balance, reducing sneezing frequency, nasal mucosal thickness, and levels of anti-OVA-IgE, autophagy-related factors (Beclin1, LC3II/LC3I), IL-17A, and retinoic acid receptor-related orphan receptor γt (RORγt), while increasing anti-OVA-IgG2a, IL-10, and Foxp3 levels (41, 42). In an AR rat model, luteolin promoted the downregulated levels of Th1-type cytokines (IL-2, IFN-γ) and suppressed the upregulated levels of Th2-type cytokines (IL-4, IL-5, IL-13), indicating its role in modulating the Th1/Th2 balance (43). Furthermore, luteolin could also affect the follicular helper T (Tfh)/follicular regulatory T (Tfr) balance (17). It reduced thyroxine T4 and thyrotropin-receptor antibody (TRAb) levels and facilitated recovery from thyroid damage in Graves disease (GD) mice. It effectively alleviated oxidative stress and restored the abnormal proportions of Tfh/Tfr and Tfh/Treg, along with regulating related mRNA levels of IL-21, Bcl-6, and Foxp3 (17). Therefore, luteolin has a significant impact on the balance and function of T cell subsets, especially in promoting Treg differentiation and regulating the Th17/Tregs, Th1/Th2, and Tfh/Tfr balances, which is beneficial for maintaining immune homeostasis in various disease conditions.

### 2.2 Inhibition of T cell activation and proliferation

T cell activation and proliferation are key events in immune responses, and inappropriate activation can lead to autoimmune diseases or excessive immune reactions (44). Luteolin has been shown to inhibit antigen-specific T cell responses (45). Verbeek and co-workers evaluated the inhibitory effects of various flavonoids on antigen-specific proliferation and IFN-γ production by human (specific for alpha B-crystallin) and murine (specific for encephalitogenic proteolipid protein [PLP] peptide) autoreactive T cells. The flavones apigenin and luteolin were strong inhibitors of both murine and human T cell responses, with antigen-specific IFN-γ production reduced more than T cell proliferation. This suggests that luteolin can target the potentially pathogenic functions of autoreactive T cells involved in autoimmune diseases (45). Moreover, luteolin suppressed T cell activation in specific models (46). Kempuraj et al. found that luteolin pretreatment inhibited myelin basic protein-induced human mast cell activation and mast cell-dependent stimulation of Jurkat T cells. In this study, mast cells activated Jurkat cells, and this interaction was inhibited by luteolin, suggesting its potential in treating autoimmune diseases related to T cell activation (46). The effect of luteolin in psoriasis was previously explored in a mouse model, and the results showed that it alleviated skin tissue lesions and symptoms. The underlying mechanisms involved suppression of IFN-γ secretion and reductions in the proportion of Th1/Th2 and Th17/Treg cells, and inhibition of increases in Th1 and Th17 cells in the peripheral blood, indicating its role in suppressing T cell activation and function in the context of psoriasis (13). These studies indicate that luteolin can effectively inhibit T cell activation and proliferation in different models, which may be useful in treating autoimmune and other immune-related diseases.

### 2.3 Enhancement of T cell-related antitumor and anti-infective immunity

T cells are crucial for the body’s defenses against tumors and infectious agents. Luteolin can enhance the antitumor and anti-infective functions of T cells (47–51). Tian et al. investigated luteolin as an antitumor vaccine adjuvant in a B16F10 mouse model and found that it activated the PI3K-AKT pathway in antigen-presenting cells (APCs), induced the activation of APCs, enhanced cytotoxic T lymphocyte (CTL) responses, and inhibited tolerogenic T cell responses. The survival rate of tumor-bearing mice was significantly improved by the adoptive transfer of CD8^+^ T cells from luteolin-immunized mice (47). In recent studies, luteolin was reported to inhibit the proliferation and invasion of colon and lung cancer cells, directly or by enhancing T cell-mediated killing pathways (48, 51). When combined with activated CTLs, it upregulated CD25 and CD69 expression on effector cells, increased the secretion of IL-2, tumor necrosis factor-α (TNF-α), and IFN-γ *in vitro*, and significantly curbed subcutaneous tumor growth and extended the survival time of tumor-bearing mice *in vivo* (48). The antitumor effect of luteolin was also investigated in H22 tumor-bearing mice, in which luteolin effectively constrained tumor-cell growth and enhanced T cell activation, cell chemotaxis, and cytokine production. It maintained a high ratio of CD8^+^ T lymphocytes in the spleen, peripheral blood, and tumor tissues, restored the cytotoxicity of tumor-infiltrating CD8^+^ T lymphocytes, and enhanced the antitumor effect when combined with the PD-1 inhibitor (49). Moreover, evidence shows that luteolin could boost the anti-infective immunity. Vaccine efficacy and long-term immune memory are critically dependent on central memory T (TCM) cells, whereas effector memory T (TEM) cells are important for clearing acute infections. Singh et al. demonstrated that, as a plant-derived Kv1.3 K^+^ channel inhibitor, luteolin promoted TCM cells by selectively inhibiting TEM cells in mice vaccinated with Bacillus Calmette-Guérin (BCG), significantly enhancing BCG vaccine efficacy (50). The same group subsequently showed that, when administered with isoniazid, luteolin promoted anti-tuberculosis (TB) immunity, reduced TB treatment duration, and prevented disease relapse. It also enhanced long-term anti-TB immunity by promoting TCM cell responses and the activity of NK and natural killer T (NKT) cells (52). Therefore, luteolin can promote CTL activation and proliferation in the antitumor context and modulate T cell memory subsets and other immune cells to enhance immune response against infectious diseases.

## 3 The effects of luteolin on NK cells

NK cells play a crucial role in the immune system, being capable of identifying and eliminating tumor cells and virus-infected cells without prior sensitization (53). Luteolin has been investigated for its impact on NK cells in multiple studies, revealing its potential to modulate immune responses related to NK cell functions (52, 54, 55). As mentioned previously, luteolin could enhance the activities of NK and NKT cells, both of which exhibit antitubercular attributes (52). Another study showed that thermal treatment of luteolin-7-O-β-glucoside, a related compound, improved its ability to augment NK cell activities, along with enhancing splenocyte proliferation and antioxidant capacity (55). Additionally, luteolin significantly enhanced NK cell activity against sensitive tumor cells (K562 line) and promoted lipopolysaccharide (LPS)-stimulated splenocyte proliferation and humoral immune responses (54). However, one study indicated that, while various flavonoids were tested, pre-treatment of Burkitt’s lymphoma cells with luteolin did not change levels of NKG2D ligands on tumor cells or the subsequent NK cell lytic activity against these cells, unlike naringenin, which enhanced NK cell cytolytic activity by increasing the expression of NKG2D ligands (56). Thus, most studies suggest that luteolin generally has positive effects on NK cell functions, such as enhancing activity and promoting related immune responses, although one study showed no impact on a specific aspect of NK cell cytotoxic activity in a particular cancer cell model. The differential effects of luteolin on NK cell-mediated tumor elimination across distinct models may reflect variations in experimental conditions, tumor microenvironment characteristics, and NK cell phenotypic states. Overall, the evidence points to luteolin’s potential as an immunomodulatory agent related to NK cells. However, further research is needed to fully understand the mechanisms and optimize its use in immunotherapy.

## 4 The effects of luteolin on dendritic cells

DCs are crucial APCs that play a central role in the initiation and regulation of immune responses (57). Luteolin has been investigated regarding its impact on DCs, shedding light on its potential to modulate immune function (16, 58). In bone marrow-derived DCs (BMDCs), it not only inhibited LPS-induced IκB phosphorylation and IκB kinase (IKK) activity but also reduced the expression levels of NF-κB, IL-12, and TNF-α (58). Intraperitoneal administration of luteolin significantly inhibited LPS-induced NF-κB expression in peripheral blood mononuclear cells and splenocytes isolated from transgenic mice (58). When combined with paclitaxel in dual-functional liposomes for esophageal cancer therapy, luteolin significantly activates the tumor microenvironment by promoting the maturation of DCs (59). Another research focused on GLM, a luteolin derivative (16). GLM treatment downregulated pro-inflammatory cytokine production (TNF-α, IL-6, IL-12p70), surface molecule expression (CD80, CD86, MHC-II), and antigen-presenting ability of the MHC-II complex in LPS-activated DCs. Importantly, its anti-inflammatory effect was dependent on MAPK/NF-κB signaling pathways. *In vivo*, GLM exerted a protective effect in dextran sulfate sodium (DSS)-induced colitis models by decreasing Th1, Th2, and Th17 cells and myeloperoxidase (MPO) levels (a marker of neutrophil infiltration) (16). Therefore, luteolin can modulate DCs by interfering with key signaling pathways related to inflammation (e.g., NF-κB pathway), while GLM further demonstrates anti-inflammatory effects on DCs. These findings suggest that luteolin and its derivatives hold promise for applications in immunomodulation and potentially in the treatment of diseases where DC-mediated immune responses are dysregulated.

## 5 The effects of luteolin on macrophages

**Figure 2:**
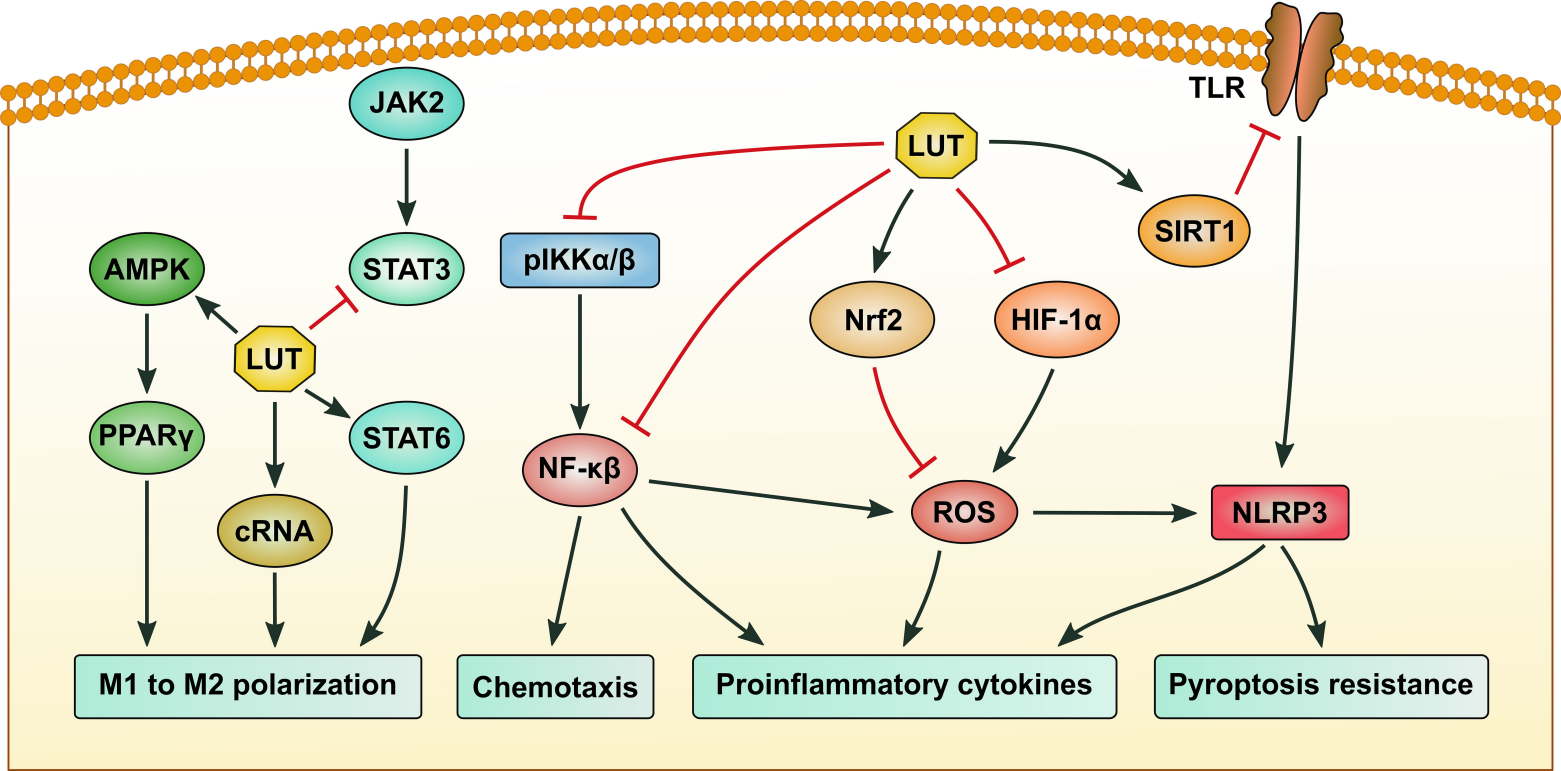
The signaling pathways regulated by luteolin in macrophages. Luteolin (LUT) suppresses M1 and promotes M2 macrophage polarization by activating the AMP-activated protein kinase (AMPK)/peroxisome proliferator-activated receptor γ (PPARγ) and signal transducer and activator of transcription 6 (STAT6) pathways, while upregulating the expression of circular RNA hsa_circ_0001326 and inhibiting STAT3 activation. LUT inhibits the production of proinflammatory cytokines by repressing nuclear factor-κB (NF-κB) and NOD-like receptor family pyrin domain-containing 3 (NLRP3) activation, as well as reactive oxygen species (ROS) generation. Inhibition of NF-κB also contributes to blocking the chemotaxis of macrophages. Suppression of the SIRT1/toll-like receptor (TLR)/NLRP3 axis can alleviate macrophage pyroptosis. Black arrows indicate promotion, while red stop symbols indicate inhibition.

Macrophages are a crucial component of the immune system, playing diverse roles in both innate and adaptive immunity. They can rapidly respond to various stimuli, making them key players in maintaining tissue homeostasis, fighting infections, and influencing the disease’s progression (60). Luteolin exerts profound immunomodulatory effects on macrophages, encompassing polarization, inflammatory signaling, and functional activities. By targeting signal transducer and activator of transcription (STAT), NF-κB, NOD-like receptor family pyrin domain-containing 3 (NLRP3), and toll-like receptor (TLR) pathways, luteolin curbs excessive inflammation while promoting tissue repair (**Figure 2**).

### 5.1 Regulation of macrophage polarization

Macrophages dynamically polarize into pro-inflammatory (M1) or anti-inflammatory (M2) phenotypes, regulated by multiple signaling pathways. STAT3 activation promotes pro-inflammatory cytokine production, while STAT6 drives M2 polarization and anti-inflammatory mediator release; their reciprocal regulation forms a core inflammatory balance axis (61). Peroxisome proliferator-activated receptor γ (PPARγ) not only directly enhances M2 polarization (synergizing with STAT6) but also intersects with the TLR4-NF-κB pathway by suppressing IκB phosphorylation. Together, these molecules and their cross-talk contribute to the restoration of immune homeostasis (62). Luteolin treatment could reduce M1 markers (iNOS, IL-1β, TNF-α, CD86) and upregulate M2 markers (Arginase 1, IL-10, IL-13, CD163, CD206) in LPS/IFN-γ-stimulated macrophages via STAT3 inhibition and STAT6 activation (63). Similarly, Yang et al. found that luteolin suppressed inflammation in DSS-induced colitis *in vivo* and promoted M2 polarization by activating the AMP-activated protein kinase (AMPK)/PPARγ pathway, reducing M1-associated cytokines (TNF-α, IL-6, and IL-1β) (14). This effect is further corroborated in THP-1-derived macrophages, where luteolin upregulated the circular RNA hsa_circ_0001326 to suppress M1 markers and enhance M2 markers (64). Collectively, these findings suggest luteolin reprograms macrophages to an anti-inflammatory phenotype through STAT and PPARγ-dependent pathways.

### 5.2 Inhibition of inflammatory signaling pathways

Luteolin potently suppresses pro-inflammatory signaling cascades in macrophages. Xue et al. reported that luteolin blocked NF-κB activation by inhibiting IKKα/β phosphorylation, thereby reducing pro-inflammatory cytokines (IL-6, TNF-α) in LPS-stimulated macrophages and ameliorating DSS-induced colitis (65). Zou et al. extended these findings; luteolin was found to exert a preventive effect on THP-1 macrophage pyroptosis by inhibiting NLRP3 inflammasome activation, largely by suppressing ROS production via nuclear factor erythroid 2-related factor 2 (Nrf2) activation as well as NF-κB inactivation. These findings suggest that luteolin has the potential to prevent pyroptosis by regulating the Nrf2-NF-κB crosstalk (66). Additionally, luteoloside reduced hepatic fibrosis by activating the TLR2/TLR4 axis, which suppressed NLRP3 inflammasome activation, secretion of pro-inflammatory cytokines (IL-1β, IL-6), and extracellular matrix deposition in macrophages and hepatic stellate cells (67). Analogously, luteolin attenuated lupus nephritis by inhibiting the hypoxia-inducible factor-1α (HIF-1α) pathway in macrophages, thereby reducing oxidative stress, inflammatory mediator release (e.g., ROS, TNF-α), and renal damage (68). These studies converge on luteolin’s ability to dampen multiple inflammatory axes, including NF-κB, NLRP3, and TLR pathways, highlighting its potential as a therapeutic agents for autoimmune, inflammatory, and fibrotic diseases.

### 5.3 Modulation of macrophage functional activities

Luteolin exerts multifaceted effects on macrophage functional activities, including phagocytosis and chemotaxis (65, 69, 70). It was reported that luteolin enhanced macrophage phagocytosis by blocking CD47 pyroglutamation via glutaminyl-peptide cyclotransferase-like protein (isoQC), disrupting the “don’t eat me” signal in tumor cells (69). Luteolin also suppressed NLRP3 inflammasome activation and TLR4/NF-κB signaling in macrophages, reducing pro-inflammatory cytokine release and promoting apoptotic cell clearance in fibrosis and sepsis (65). Regarding chemotaxis, luteolin inhibited CCL2-induced macrophage migration by antagonizing IKKα/β phosphorylation and NF-κB nuclear translocation, reducing inflammatory infiltration in colitis (65). In periodontitis, luteolin ameliorated periodontal inflammation and bone loss by modulating mitochondrial dynamics, promoting M2 macrophage polarization, and suppressing the JAK2/STAT3 signaling pathway (71). Collectively, these findings position luteolin as a promising therapeutic for macrophage-driven diseases by enhancing phagocytic clearance while dampening chemotaxis and inflammation.

### 5.4 Synergistic effects and therapeutic potential

Luteolin often acts synergistically with other compounds to augment anti-inflammatory effects. Notably, in LPS-stimulated RAW264.7 macrophages, the expression of ROS, NO, TNF-α, IL-6, and IL-1β was significantly increased, which was reversed by the combination of luteolin and paeoniflorin via suppression of the NF-κB/MAPK signaling pathway (72). Similarly, in hepatitis B virus infection, luteolin enhanced cGAS-STING activation in macrophages when paired with schisandrin C to control viral replication (73). These combinatorial strategies highlight luteolin’s versatility in therapeutic regimens.

## 6 The effects of luteolin on neutrophils

**Figure 3:**
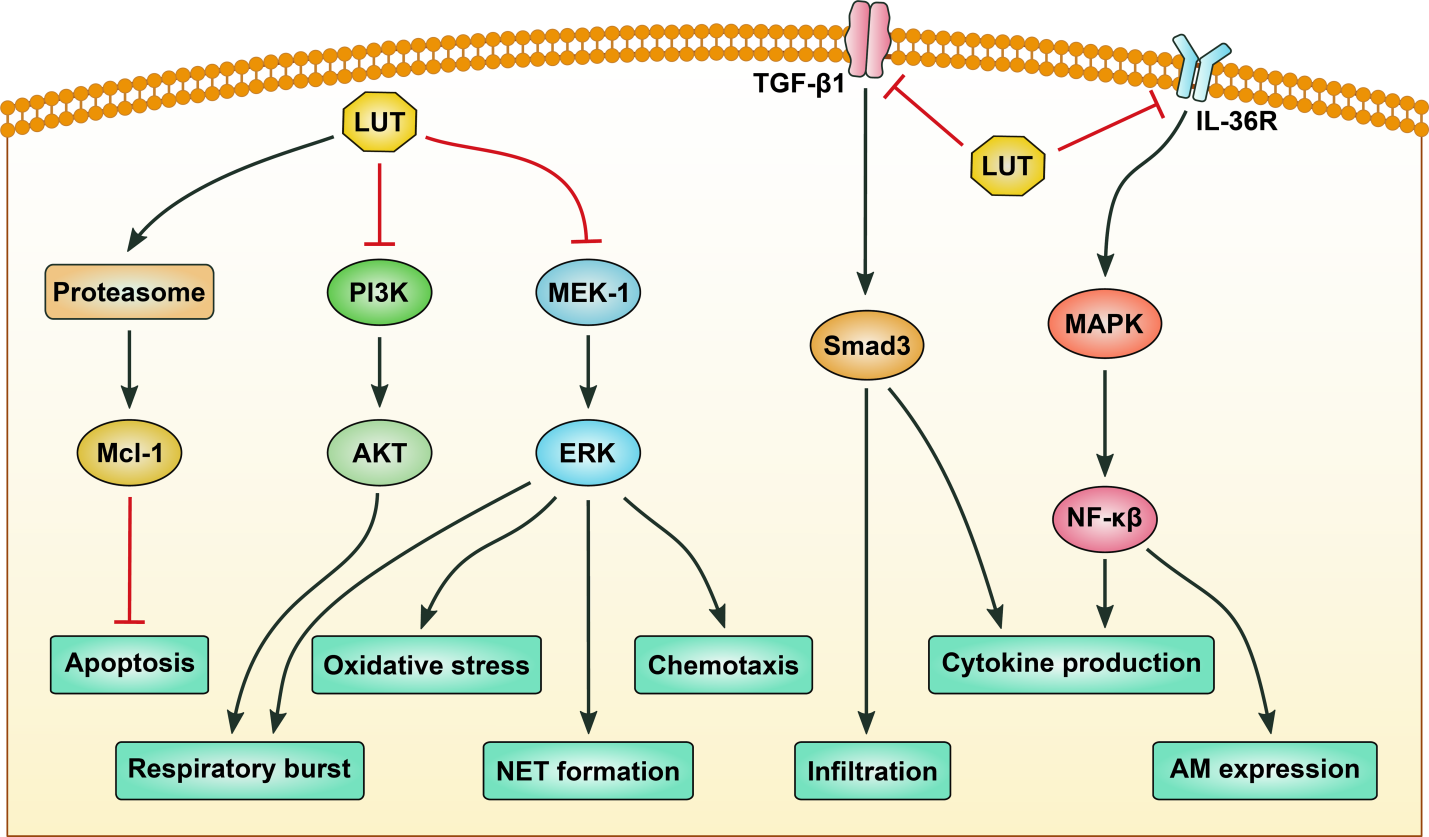
The signaling pathways regulated by luteolin in neutrophils. Luteolin (LUT) induces apoptosis of neutrophils by activating proteasome-mediated anti-apoptotic protein Mcl-1 degradation. LUT inhibits respiratory burst of neutrophils by targeting phosphatidylinositol 3-kinase (PI3K)/protein kinase B (AKT) and mitogen-activated protein kinase kinase-1 (MEK-1)/extracellular signal-regulated kinase (ERK) signaling pathways. Inhibition of MEK-1/ERK signaling also contributes to suppression of oxidative stress, chemotaxis, and neutrophil extracellular trap (NET) formation of neutrophils. Infiltration and proinflammatory cytokine production of neutrophils are repressed by LUT via blocking transforming growth factor-β1 (TGF-β1)/Smad3 signaling. Production of proinflammatory cytokines and expression of adhesion molecules are inhibited by LUT via modulating the mitogen-activated protein kinase (MAPK)/nuclear factor-κB (NF-κB) signaling pathway. Black arrows indicate promotion, while red stop symbols indicate inhibition.

Neutrophils are key effector cells in innate immunity, mediating inflammatory responses through mechanisms such as ROS production, neutrophil extracellular trap (NET) formation, and protease release via a series of cellular signaling pathways (74). Notably, the MAPK/NF-κB pathway in neutrophils belongs to a broader danger-sensing network: upon detecting pathogens (via TLR) or tissue damage (via damage-associated molecular patterns), activation of MAPK phosphorylates NF-κB, triggers neutrophil degranulation and NET formation. It also crosstalks with the PI3K/AKT axis to coordinate neutrophil recruitment and functions (75, 76). Luteolin exerts pleiotropic effects on neutrophils, primarily by inhibiting oxidative stress, inflammatory signaling (MAPK/NF-κB), and adhesion molecule expression, while promoting apoptosis (**Figure 3**). These properties make it a promising candidate for treating neutrophilic inflammatory diseases such as arthritis, asthma, and ALI. However, inconsistencies in structure-activity relationships and *in vivo* outcomes underscore the need for further mechanistic studies and translational research to optimize its therapeutic potential.

### 6.1 Regulation of neutrophil oxidative stress and adhesion

Neutrophils contribute to inflammation through oxidative stress, protease release, and adhesion molecule-mediated tissue infiltration. In inflammatory arthritis models, luteolin inhibited neutrophil superoxide anion generation, ROS production, and NET formation by targeting the Raf1/mitogen-activated protein kinase kinase-1 (MEK-1)/extracellular signal-regulated kinase (ERK) signaling axis, thereby reducing leukocyte infiltration and paw edema (77). Similarly, in an endotoxin-induced ALI model, luteolin attenuated neutrophilic inflammation by scavenging ROS, reducing myeloperoxidase activity, and inhibiting MAPK/NF-κB-mediated secretion of TNF-α and intercellular adhesion molecule-1 (ICAM-1) (78). Mechanistically, luteolin can inhibit cyclic adenosine monophosphate (cAMP)-phosphodiesterases (PDEs) activity and the expression of lymphocyte function-associated antigen-1 in neutrophils (79). Moreover, luteolin metabolites, such as luteolin monoglucuronide, are hydrolyzed by beta-glucuronidase released from neutrophils at inflammatory sites to yield free luteolin, which suppresses TNF-α-induced ICAM-1 expression and interferes with adhesion between neutrophils and endothelial cells (80). Thus, these findings indicate that luteolin can mitigate neutrophil-driven oxidative stress and inflammation by targeting redox signaling, adhesion molecules, and inflammatory mediator release.

### 6.2 Regulation of neutrophilic signaling pathways

Neutrophils, key effector cells in acute inflammation, contribute to tissue damage through chemotaxis, respiratory burst, and delayed apoptosis. Luteolin modulates neutrophil functions via distinct signaling mechanisms. In LPS-induced ALI, luteolin attenuated neutrophil chemotaxis and respiratory burst by inhibiting MEK/ERK and PI3K/AKT phosphorylation, thereby reducing leukocyte infiltration and protein extravasation (12). Similarly, luteolin blocked Fcγ receptor-mediated respiratory burst in granulocytes by targeting downstream signaling cascades, though this effect did not translate to blister suppression in pemphigoid models, suggesting context-dependent efficacy (81). Mechanistically, luteolin induced neutrophil apoptosis through proteasomal degradation of Mcl-1, a pro-survival Bcl-2 family protein, thereby overcoming survival signals from LPS or granulocyte-macrophage colony-stimulating factor (GM-CSF). This apoptotic effect is caspase-dependent and enhanced in inflammatory microenvironments, positioning luteolin as a pro-resolution agent (82). Taken together, these studies suggest luteolin’s multifaceted regulation of neutrophil signaling, spanning MAPK/PI3K pathways, Fcγ receptor responses, and apoptotic machinery.

### 6.3 Regulation of neutrophilic inflammation

Neutrophilic inflammation drives tissue damage in diseases such as lung fibrosis, asthma, and periodontitis, making modulation of neutrophil activation a therapeutic priority. Luteolin demonstrates efficacy across these contexts through distinct mechanistic pathways. In bleomycin-induced lung fibrosis, luteolin reduced neutrophil infiltration and inflammatory cytokines (TNF-α, IL-6) in the BALF while inhibiting TGF-β1/Smad3-mediated myofibroblast differentiation and epithelial-to-mesenchymal transition (83). This dual anti-inflammatory and antifibrotic effect translates to reduced collagen deposition and preserved lung architecture (83). In neutrophilic asthma models, luteolin suppressed IL-36γ secretion and MAPK/IL-1β signaling, attenuating neutrophilic airway inflammation and hyperresponsiveness (84). Mechanistically, luteolin blocked LPS-induced IL-36γ upregulation in bronchial epithelial cells, thereby disrupting the pro-inflammatory feedback loop, thus reducing the secretion of inflammatory factors and alleviating the inflammatory response (84). In periodontitis, luteolin mitigated LPS-induced alveolar bone loss by inhibiting neutrophil infiltration, NF-κB activation, and the expression of pro-inflammatory enzymes (iNOS, COX-2) and cytokines (IL-6, TNF-α), while preserving collagen integrity (85). Collectively, these studies highlight luteolin’s ability to target neutrophilic inflammation through redox regulation, cytokine modulation, and extracellular matrix preservation, positioning it as a promising candidate for neutrophil-driven inflammatory disorders.

## 7 The effects of luteolin on eosinophils

Eosinophils are critical effector cells in allergic inflammation, mediating tissue damage through cytotoxic granule release and pro-inflammatory cytokine production (86). Luteolin exerts multifaceted effects on eosinophils, primarily by inhibiting their recruitment, activation, and survival; dampening Th2 cytokine production; and promoting anti-inflammatory Tregs. These properties position luteolin as a promising candidate for treating eosinophil-driven diseases like asthma and allergic rhinitis.

### 7.1 Inhibition of eosinophil activation and recruitment

Numerous studies have demonstrated that luteolin can reduce eosinophil infiltration in allergic models (87–89). In murine models of allergic asthma, luteolin reduced eosinophil and neutrophil infiltration in BALF, concomitant with decreased levels of IL-4, IL-5, and IL-13 in lung homogenates (87). This effect was paralleled in allergic nasal inflammation, where luteolin attenuated eosinophilic infiltration, mucus hypersecretion, and serum house dust mite-specific IgE, while downregulating CD4^+^ IL-4-secreting T cells via inhibition of STAT6/GATA3 signaling (88). It suppressed prostaglandin E2 (PGE2) production and Th2 cytokine transcription in both *in vitro* and *in vivo* settings (88). In OVA-induced asthma, luteolin-7-O-glucoside (L7G), a bioactive derivative, dose-dependently reduced eosinophil infiltration and PGE2 levels in BALF, while inhibiting IL-4, IL-5, and IL-13 mRNA expression (89). Lee et al. evaluated the inhibitory effects of luteolin on the immediate-phase asthmatic response (IAR) and the late-phase asthmatic response (LAR) to aerosolized OVA exposure in conscious OVA-sensitized guinea pigs. The results showed that the anti-asthmatic effect of luteolin in IAR and LAR likely reflects inhibition of eosinophil recruitment that underlies the subsequent pathological changes and suppression of biochemical mediators such as histamine, phospholipase A2, and eosinophil peroxidase in the asthmatic lung, thereby reducing antigen-induced bronchoconstriction (90). These effects were attributed to luteolin’s ability to interfere with eosinophils’ recruitment and activation, thereby disrupting the inflammatory cascade.

### 7.2 Mitigation of eosinophilic inflammation via modulating Th2 cells and Tregs

Luteolin modulates eosinophil-driven inflammation by targeting Th2 cytokines and transcription factors. Park et al. reported that luteolin-4’-O-glucoside suppressed IL-5 bioactivity, a key eosinophil chemoattractant and growth factor, with an IC_50_ of 3.7 μM (91). A recent study revealed that luteolin reduced IL-4, IL-5, and IL-13 levels in mice with eosinophilic chronic rhinosinusitis (ECRS), restoring olfactory sensory neurons via TLR4/NF-κB pathway inhibition (18). Mechanistically, luteolin inhibits STAT6 phosphorylation and GATA3 expression, both critical for Th2 differentiation (18). In allergic rhinitis models, luteolin decreases CD4^+^ IL-4-secreting T cells by downregulating STAT6/GATA3 signaling, thereby reducing eosinophil infiltration and mucus hypersecretion (88). This finding aligns with another study where luteolin induced CD4^+^CD25^+^ Tregs expressing Foxp3 and TGF-β1, counteracting Th2 dominance and reducing eosinophil recruitment (40). Accordingly, luteolin’s dual actions—suppressing Th2 cytokine secretion and promoting Tregs—offer a multifaceted approach to allergic inflammation.

### 7.3 Antioxidant and anti-apoptotic effects of luteolin in eosinophilic inflammation

Luteolin exhibits robust antioxidant and anti-apoptotic properties in eosinophilic disorders by targeting redox imbalance and apoptotic signaling. In the ECRS mouse model, luteolin reduced oxidative stress in olfactory sensory neurons (OSNs) by increasing the activities of antioxidant enzymes (Superoxide Dismutase [SOD], catalase [CAT], glutathione peroxidase [GSH-Px]) and decreasing malondialdehyde (MDA), while attenuating OSN apoptosis by Bcl-2 upregulation and caspase-3/9 inhibition. The anti-apoptotic effects of luteolin on OSNs were reversed by LPS-induced TLR4/NF-κB activation, suggesting that luteolin exerts its anti-apoptotic effects by inhibiting TLR4/NF-κB (18). These effects were validated in human olfactory epithelial cells, where luteolin reversed LPS-induced ROS generation and apoptosis (18). The dual antioxidant and anti-apoptotic actions of luteolin in eosinophilic inflammation highlight its therapeutic potential for diseases marked by oxidative damage and excessive cell death.

## 8 The effects of luteolin on basophils

Basophils play a central role in IgE-mediated allergic inflammation by releasing histamine, leukotrienes (LT), and cytokines (92). Studies have shown that luteolin is a promising modulator of basophil function, primarily through its anti-inflammatory and immunoregulatory properties (93–95). It potently suppresses the release of allergic mediators from basophils; in parallel, luteolin dose-dependently inhibits histamine, LT, prostaglandin D2 (PGD2), and GM-CSF release from human cultured mast cells (HCMCs), with IC_50_ values comparable to quercetin and baicalein (93). Mechanistically, luteolin blocks Ca²^+^ influx and protein kinase C (PKC) translocation, critical steps in IgE-mediated activation (93). Similarly, luteolin inhibits A23187- and phorbol myristate acetate (PMA)-induced CD40 ligand expression in KU812 cells (a human basophilic cell line), and suppresses histamine release by activated basophils (94). These effects are linked to suppressed AP-1 transcription factor activation, as demonstrated by reduced c-Jun phosphorylation and deoxyribonucleic acid (DNA) binding (95). This activity is conserved across species: 6-methoxyluteolin from Chrysanthemum zawadskii downregulated FcϵRI α chain expression in KU812 cells, thereby reducing histamine release and Ca²^+^ influx (96). Additionally, luteolin inhibits degranulation of KU812 cells and the release of IL-6 and TNF-α induced by ω-5 gliadin. In the Caco-2 cell monolayer, it inhibited zonulin release, and significantly increase the expression of tight junction proteins (Occludin and ZO-1), which implicates that luteolin may be used to alleviate food allergic reactions and intestinal inflammation (97). The anti-allergic effect of luteolin was supported by another study, where luteolin-rich olive oil emulsions inhibited β-hexosaminidase release and gene expression related to type I allergy in a rat basophilic leukemia RBL-2H3 cells (98). Furthermore, it was identified as a bioactive component in *adlay bran* extract that reduced the release of histamines and cytokines and suppressed the production of AKT in RBL-2H3 cells, thereby revealing the mechanisms of the anti-allergic effects of *adlay* (99). Luteolin targets Th2 cytokines that drive allergic inflammation. It inhibits IL-4 and IL-13 synthesis in activated human basophils, with an IC_50_ of 2−5 μM (100). Notably, studies suggest structural determinants of luteolin’s efficacy. The 5,7,3’,4’-tetrahydroxyflavone structure of luteolin is critical for inhibiting CD40 ligand expression, with methylation at the 4’-position abolishing activity (94). Watanabe et al. further showed that luteolin inhibits both early (Ca²^+^/PKC) and late (MAPK) signaling steps in RBL-2H3 cells, distinguishing it from coumarin derivatives (101). In conclusion, luteolin exerts multifaceted effects on basophils, including inhibition of mediator release, cytokine production, and CD40 ligand expression, alongside barrier-protective actions. These properties position luteolin as a promising candidate for basophil-mediated allergic disease therapy.

## 9 Limitations of current evidence in extrapolating to humans

Despite extensive preclinical data supporting luteolin’s immunomodulatory effects on immune cells, several limitations hinder translation to humans. First, there are critical species differences: all studies use murine models, but murine and human immune systems differ in cell subset ratios, cytokine signaling, and tissue responses. Second, dose variability is problematic: preclinical studies employ a wide range of luteolin doses (0.1–200 mg/kg/day) without standardization (**Table 1**), complicating the determination of safe and effective doses for human clinical trials. Third, overreliance on single-model systems and limited reproducibility constrains generalizability: most findings come from one specific model with few cross-model validations or independent replication experiments, raising concerns that observed effects may be context-dependent rather than robust. These limitations create significant pitfalls when extrapolating preclinical results to humans, so future research should prioritize human cell-based models, standardized dosing protocols, and multi-model testing to reduce the risk of failed clinical trials.

## 10 Summary and future direction

**Figure 4:**
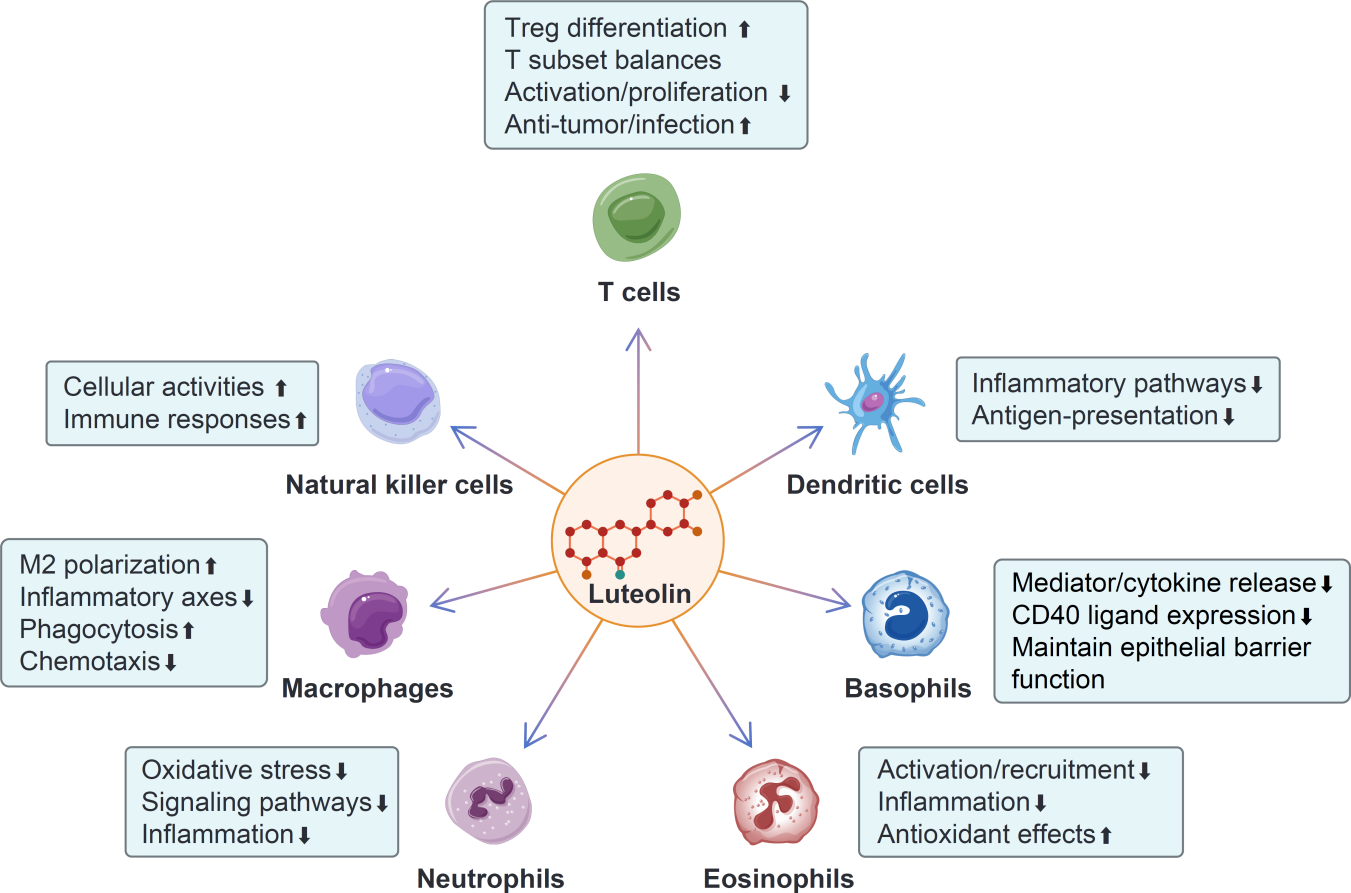
The modulatory effects of luteolin on different immune cells. Luteolin promotes Treg differentiation, enhances the anti-tumor and anti-infection effects of T cells, and inhibits T cell activation and proliferation. Additionally, it modulates the balances among different T cell subsets. Luteolin enhances the activities and immune responses of natural killer (NK) cells. It inhibits the inflammatory pathways and antigen-presentation of dendritic cells (DCs). Luteolin promotes M2 macrophage polarization and phagocytosis, while inhibiting the inflammatory axes and chemotaxis of macrophages. It inhibits oxidative stress, signaling pathways, and neutrophil-mediated inflammation. Luteolin suppresses the activation, recruitment, and inflammation of eosinophils, while enhancing their anti-oxidant effect. Moreover, luteolin inhibits the release of mediators and cytokines from basophils, as well as CD40 ligand expression, and promotes the maintenance of barrier integrity function.

Luteolin has emerged as a versatile immunomodulatory agent with profound effects on multiple immune cell populations, including T cells, NK cells, DCs, macrophages, neutrophils, eosinophils, and basophils (**Figure 4**). Its pleiotropic actions are underpinned by the regulation of key signaling pathways (e.g., NF-κB, MAPK, STAT, and PPARγ), modulation of cell polarization and activation, and restoration of immune homeostasis in preclinical models of inflammation, infection, and cancer. Despite the promising potential of luteolin as an immunomodulatory agent, several significant challenges remain. A major hurdle is the design of clinically appropriate drug formulations. Luteolin is a lipophilic compound that poses challenges in terms of its solubility and bioavailability. An ideal formulation should not only ensure effective bioavailability and therapeutic efficacy but also minimize the adverse reactions associated with luteolin or its excipients. Nanoparticles, liposomes, or other advanced delivery systems can be employed to improve the solubility and targeted delivery of luteolin (102). Various nanoparticles were prepared, including twelve types of lipid-polymer hybrid nanoparticles, protein-polysaccharide composite nanoparticles, and nanoemulsions. These delivery systems improve aqueous solubility and bioavailability, exhibit prolonged drug release, enhance tissue permeability, and improve therapeutic efficacy in animal models of various diseases (103–105). Furthermore, conflicting results have been observed regarding the effects of luteolin on the same immune cell populations across different disease models. Although a dose-dependent inhibitory effect of luteolin on T cell activation and proliferation has been observed *in vitro*, high-dose luteolin (200 mg/kg) can promote T cell activation and infiltration in cancer models, whereas low-dose luteolin (50 mg/kg) inhibits T cell activation and function in autoimmune disease models. Therefore, these results suggest that the effects of luteolin on immune cells depend on the disease context. By clarifying how luteolin interacts with different signaling pathways and cellular components, we can better optimize clinical translation. For instance, gene editing could be employed to elucidate the molecular mechanisms underlying luteolin’s actions. In addition, the application of luteolin in human-derived disease models and multi-omics studies remains limited. Human-derived disease models, such as patient-derived xenografts (PDXs) and organoids, can provide more physiologically relevant information than traditional animal models. Moreover, multi-omics approaches, including single-cell transcriptomics, proteomics, and metabolomics, can offer a comprehensive view of the molecular changes induced by luteolin. By addressing issues related to drug formulation, administration regimens, molecular mechanisms, and the use of human-derived models and multi-omics approaches, we can unlock the full potential of luteolin as a therapeutic agent for a wide range of immune-related disorders and beyond. This may not only benefit patients with immune-related diseases but also contribute to the development of innovative therapeutic strategies in immunology.

## Glossary

ROS: reactive oxygen species
COX-2: cyclooxygenase-2
iNOS: inducible nitric oxide synthase
NF-κB: nuclear factor-κB
MAPK: mitogen-activated protein kinase
PI3K: phosphatidylinositol 3-kinase
AKT: protein kinase B
NK cells: natural killer cells
DCs: dendritic cells
Tregs: regulatory T cells
CLP: cecal ligation puncture
ALI: acute lung injury
BALF: bronchoalveolar lavage fluid
TGF-β1: transforming growth factor-β1
OVA: ovalbumin
Th: T helper
IFN-γ: interferon-γ
Tfh: follicular helper T
Tfr: follicular regulatory T
TRAb: thyrotropin-receptor antibody
GD: Graves disease
APCs: antigen-presenting cells
CTL: cytotoxic T lymphocyte
TNF-α: tumor necrosis factor-α
TCM: central memory T
TEM: effector memory T
BCG: Bacillus Calmette-Guérin
TB: tuberculosis
NKT cells: natural killer T cells
LPS: lipopolysaccharide
BMDCs: bone marrow derived DCs
IKK: IκB kinase
DSS: dextran sulfate sodium
MPO: myeloperoxidase
STAT: signal transducer and activator of transcription
NLRP3: NOD-like receptor family pyrin domain-containing 3
TLR: toll-like receptor
AMPK: AMP-activated protein kinase
PPARγ: peroxisome proliferator-activated receptor γ
NET: neutrophil extracellular trap
MEK-1: mitogen-activated protein kinase kinase-1
ERK: extracellular signal-regulated kinase
ICAM-1: intercellular adhesion molecule-1
cAMP: cyclic adenosine monophosphate
PDEs: phosphodiesterases
HIF-1α: hypoxia-inducible factor-1α
L7G: luteolin-7-O-glucoside
IAR: immediate-phase asthmatic response
LAR: late-phase asthmatic response
ECRS: eosinophilic chronic rhinosinusitis
OSNs: olfactory sensory neurons
SOD: Superoxide Dismutase
CAT: catalase
GSH-Px: glutathione peroxidase
MDA: malondialdehyde
PGD2: prostaglandin D2
GM-CSF: granulocyte macrophage-colony stimulating factor
HCMCs: human cultured mast cells
