# Supplementary material for: Luteolin as a multifaceted immunomodulator: insights into its effects on diverse immune cell populations and therapeutic implications
Source: Front Immunol. 2025 Oct 14;16:1621367. doi: 10.3389/fimmu.2025.1621367 (PMC12558972; doi:10.3389/fimmu.2025.1621367)
Supplement: Supplementary file 1 [file DataSheet1.pdf]

Supplemental Table 1 Pathways regulated by luteolin in different immune cells and therapeutic implications

	Key pathways	In vivo models	Therapeutic implications
Effector memory T cells	Inhibit Kv1.3 K^+ channel pathway	Tuberculosis infected mice	Inflammatory diseases
$CD8^+$ T cells	Target peroxiredoxin-2 and inhibit PD-L1 expression	Lewis-bearing mouse model	Cancers
Dendritic cells	Inhibit MAPK/NF-κB pathway	DSS-induced mouse colitis	Autoimmune diseases
	Activate PI3K/AKT pathway	B16F10-bearing mouse model	Cancers
Macrophages	Activate AMPK/PPARγ pathway	DSS-induced mouse colitis	Autoimmune diseases
	Inhibit NF-κB pathway	DSS-induced mouse colitis	Autoimmune diseases
	Inhibit HIF-1α pathway	Mouse lupus nephritis	Autoimmune diseases
	Inhibit JAK2/STAT3 pathway	Rat periodontitis	Inflammatory diseases
Neutrophils	Inhibit Raf1/MEK-1/ERK pathway	Mouse inflammatory arthritis	Inflammatory diseases
	Inhibit MAPK/NF-κB and PI3K/AKT pathways	Mouse acute lung injury	Inflammatory diseases
	Inhibit TGF-β1/Smad3 pathway	Mouse lung fibrosis	Inflammatory diseases
	Inhibit MAPK/IL-1β pathway	Mouse asthma models	Inflammatory diseases
	Inhibit NF-κB pathway	Rat periodontitis	Inflammatory diseases
Eosinophils	Inhibit STAT6/GATA3 pathway	Mouse allergic nasal inflammation	Inflammatory diseases
	Inhibit TLR4/NF-κB pathway	Eosinophilic chronic rhinosinusitis mice	Inflammatory diseases

MAPK: mitogen-activated protein kinase; NF-κB: nuclear factor-κB; PI3K: phosphatidylinositol 3-kinase; AKT: protein kinase B; AMPK: AMP-activated protein kinase; PPARγ: peroxisome proliferator-activated receptor γ; HIF-1α: hypoxia-inducible factor-1α; JAK: Janus kinase; STAT: signal transducer and activator of transcription; MEK-1: mitogen-activated protein kinase kinase-1; ERK: extracellular signal-regulated kinase; TGF-β1: transforming growth factor-β1; IL-1β: interleukin-1β; TLR4: toll-like receptor 4.
